# Thyroid diseases and second to fourth digit ratio in Polish adults

**DOI:** 10.1038/s41598-021-98436-4

**Published:** 2021-09-23

**Authors:** Paulina Pruszkowska-Przybylska, Magdalena Kobus, Aleksandra Iljin, Joanna A. Wiktorska, Elżbieta Żądzińska, Aneta Sitek

**Affiliations:** 1 Department of Anthropology, Faculty of Biology and Environmental Protection, University of Lodz, 90-237 Lodz, Poland; 2 Department of Plastic, Reconstructive and Aesthetic Surgery, Medical University of Lodz, Poland, 90-153 Lodz, Poland; 3 MelissaMed M. Gauer, General Partnership, 90-135 Lodz, Poland; 4 Biological Anthropology and Comparative Anatomy Research Unit, School of Medicine, University of Adelaide, Adelaide, South Australia 5005 Australia

**Keywords:** Endocrine system and metabolic diseases, Predictive markers

## Abstract

The association between second to fourth finger ratio and thyroid diseases is unexplained. There is a possible interaction between prenatal exposition to sex hormone and thyroid functions in the adulthood. The study included 175 adults investigated in Łódź in the central Poland. It consisted of two main parts: a survey including questions about occurrence of thyroid gland dysfunction and anthropometric measurements (body mass and height and length of the second and fourth finger, waist and hip circumferences). The women who had thyroid disease had higher 2D:4D digit ratio (left hand) (mean = 1.004; SD = 0.036) than healthy ones (mean = 0.989; SD = 0.030) (t = − 2105; *p* = 0.038; d = 0.707). The association between thyroid diseases occurrence and prenatal steroid hormone exposition is noticed. Only females who had thyroid diseases tend to have higher 2D:4D digit ratio, for left hand.

## Introduction

Estrogens and androgens are two of the five classes of the steroid hormones. Women and men have them in significantly different amounts that affect their sexual traits and physiology^1^. The widely known fingers ratio—2D:4D was identified with proportion of estrogen and androgens in prenatal life^2^, but also with current level of the sex hormones^3,4^. However, some studies pointed out that association between the second to fourth finger is only prenatal effect and current sex hormone concentration in this mechanism is questionable^5–7^.

The association between thyroid hormones and sex hormones is noticed. In the cells of thyroid tissue are located sex hormone receptors, thus thyroid diseases may be linked with level of sex hormones^8^. The thyroid is a gland which produces hormones regulating metabolism—triiodothyronine (T3) and thyroxine (T4). The thyroid regulation is under control of HPA axis. The proper hormonal balance is crucial for the body homeostasis. Inappropriate thyroid hormones concentration may stem from thyroid diseases such as overactive thyroid gland or hypothyroidism^9^.

There are also studies which linked sex hormones concentrations with the thyroid diseases. Hypothyroidism is more common among individuals with increased estrogen levels^10^, thus hypothyroidism is less frequently observed in men than in women^11^.

Additionally, thyroid cancer is associated with excess of estrogens^12–16^.

Moreover, thyroid autoimmune diseases are observed in men with increased estrogen levels^10,17,18^.

There are few studies which evaluated link between digit ratio and thyroid diseases. Tabachnik et al.^19^ pointed out that thyroid disorders were more common among women with higher 2D:4D. The prenatal programing of thyroid and sex hormones stays tentative. Wilcoxon and Redei^20^ showed in the animal studies that among adults, thyroid function is programmed in utero, however that might be triggered by sex steroids in the adulthood.

Moreover, body mass index (BMI) seems to be an important factor associated with sex hormones proportions and thyroid diseases. Female pattern of 2D:4D finger ratio is associated with elevated BMI, as presented Fink et al.^21^ among males and Bagepally et al.^22^ among both sexes. Facing the problem of thyroid dysfunctions and body mass dysregulation the direct association is uncertain. In the case of more common hypothyroidism the patients tend to have higher BMI and fat mass^23,24^. It relates to lower cell metabolism triggered by lower T4 value and leptin interaction^25^.

In this study we have examined the association between second to fourth finger ratio and thyroid diseases, due to possible sex hormone prenatal exposition and interaction with thyroid hormones in the adulthood taking into account body mass index (BMI) and waist to hip ratio (WHR).

## Material and methods

The study included 175 adults (101 females and 74 males) investigated between July 2020 and September 2020 in Łódź (population 680,000) in the central Poland. It consisted of two main parts: a survey and anthropological measurements. The survey was filled in by the probants during measurements and contained information about: occurrence of thyroid diseases and the family’ history of the thyroid diseases. Among all patients 37 declared thyroid diseases (hypothyroidism or Hashimoto’s disease), the rest 138 did not declare any thyroid diseases. The second part of the investigation including the following anthropological measurements performed according to Martin procedure^26^ by qualified staff received standardized training for each type of measurements: body weight (with an accuracy of 0.1 kg) using a scale, body height (with an accuracy of 0.001 m) using an anthropometer, waist and hip circumferences using (with an accuracy of 0.001 m) using an anthropometric tape, the length (from a midpoint of the flexure-crease proximal to the palm to the tip of the finger) of the second and the fourth digits in both hands (with an accuracy of 0.001 m) directly by hands using a sliding calliper (Vernier calliper). All measurements were repeated twice and that the average value was taken to minimize the measurements error.

Some of the measurements were used to calculate the following indexes: the 2D:4D digit ratio [the quotient of the length of the second digit and the fourth digit (mm)], the BMI—the body mass index [the quotient of body mass (kg) and the square of height (m^2^)], the WHR—the waist to hip ratio [the quotient of waist circumference (cm)] and hip waist circumference.

This study was conducted according to the guidelines of the Declaration of Helsinki, and all procedures involving research study participants were approved by the consent of the Bioethics Committee at the Medical University of Lodz (RNN/374/19/KE). Written informed consent was obtained from all participants.

### Statistical analysis

The Shapiro–Wilk test was used to verify the distribution of the continuous variables.

Finger ratio 2D:4D for right and left hand and BMI were normally distributed (box-cox transformation was used due to the skewness of BMI) thus parametric tests were applied for the further analysis.

The Spearman correlation was used to evaluate association between age, BMI, WHR versus 2D:4D finger ratio for both hands separately for healthy and ill man and women.

The Chi^2^ test was used to check frequency differences between BMI categories according to WHO among investigated individuals and frequency differences between investigated individuals who reported and those who did not report thyroid diseases in the 2D:4D digit ratio categories (below 1—male pattern, above or equal 1—female pattern) for the both hands. Odds ratio was calculated to asses if female or male hand pattern is a risk factor in thyroid diseases.

The student *t*‐tests were used to investigate the age, BMI (after Box-Cox transformation), WHR and finger ratio (right, left hand) differences depending on sex and occurrence of thyroid diseases. Additionally, student t-test for independent samples were used to verify differences between 2D:4D digit ratio for right and left hand. There were statistically significant differences between 2D:4D digit ratio for right and left hand, (t = − 2.668; *p* = 0.008), thus the further analyses were conducted separately for the right and left hand. To calculate effect size for each comparisons Cohen's d value were calculated.

Multiple regression models were applied to verify which variables importantly explain variability of the 2D:4D for both hands.

The Statistica ver. 12.0 software was used to perform all calculations.

## Results

The females who had not thyroid diseases were characterised by a lower BMI (after Box-Cox transformation) (mean = 1.034; SD = 0.008) and WHR (mean = 0.925; SD = 0.062) than the males (mean = 1.039; SD = 0.008 and mean = 0.958; SD = 0.092)) (BMI: t = − 3.640; *p* < 0.001; d = 0.625; WHR: t = − 2.440; *p* = 0.016; d = 0.420). There were not any differences between males and females regarding age, BMI, WHR, and digit ratio 2D:4D for both hands among individuals who had thyroid diseases (Table 1).

**Table 1:** Differences in age, BMI, 2D:4D (R) and 2D:4D (L) between females and males.

Sex	Variables for individuals who had not thyroid diseases						T	*p*	d
	Means	N	SD	Q25	Median	Q75			
**Age**									
Females	51.159	69	12.582	43.000	49.000	60.000	0.435	0.664	0.073
Males	50.188	69	13.905	38.000	47.000	61.000			
**BMI (after Box–Cox transformation)**									
Females	1.034	69	0.008	1.030	1.033	1.038	− 3.640	**< 0.001**	0.625
Males	1.039	69	0.008	1.033	1.038	1.043			
**WHR**									
Females	0.925	69	0.062	0.880	0.920	0.960	− 2.440	**0.016**	0.420
Males	0.958	69	0.092	0.890	0.960	1.020			
**2D4D digit ratio (right hand)**									
Females	0.980	69	0.033	0.958	0.986	1.000	0.862	0.390	0.147
Males	0.975	69	0.035	0.951	0.974	1.000			
**2D4D digit ratio (left hand)**									
Females	0.989	69	0.030	0.971	0.986	1.014	1.694	0.093	0.295
Males	0.980	69	0.031	0.963	0.986	1.000			

	Variables for individuals who had thyroid diseases								
**Age**									
Females	52.969	32	13.094	42.500	52.000	65.000	0.059	0.954	0.202
Males	55.800	5	14.923	45.000	47.000	70.000			
**BMI (after Box–Cox transformation)**									
Females	1.034	32	0.008	1.030	1.033	1.038	− 0.442	0.661	0.625
Males	1.039	5	0.008	1.033	1.038	1.043			
**WHR**									
Females	0.953	32	0.108	0.860	0.950	1.030	− 1.096	0.281	0.599
Males	1.008	5	0.072	0.950	1.010	1.040			
**2D4D digit ratio (right hand)**									
Females	0.986	32	0.038	0.961	0.985	1.021	1.604	0.118	0.693
Males	0.956	5	0.048	0.925	0.948	0.975			
**2D4D digit ratio (left hand)**									
Females	1.004	32	0.036	0.973	1.000	1.042	1.516	0.138	0.740
Males	0.977	5	0.037	0.959	0.960	1.000			

Bold indicates statistically significant results (*p* < 0.05).

The Table 2 showed that among individuals with thyroid diseases obesity and overweight were more frequent than among healthy ones. 57.97% of females without thyroid diseases had normal weight and 40.58% were overweight or obese, but among females who had thyroid diseases 37.50% had normal weight and 62.5% were overweight or obese. In the case of men without thyroid diseases 24.64% had normal weight and 73.92% were overweight or obese, but among males who had thyroid diseases 20.00% had normal weight and 80.00% were overweight or obese. Independently on thyroid diseases occurrence, men more frequently had overweight or obesity than women.

**Table 2:** Frequency distribution among investigated individuals in the BMI categories.

BMI WHO categories	Sex	Lack of thyroid diseases N (%)	Thyroid diseases N (%)	Row Totals	Chi^2^	*p*
Underweight	Female	1 (1.14)	0	1	–	–
	Male	1 (1.14)	0	1		
Total		2	0	2		
Normal weight	Female	40 (57.97)	12 (37.50)	52	2.715	0.099
	Male	17 (24.64)	1 (20.00)	18		
Total		57	13	70		
Overweight	Female	20 (28.99)	12 (37.50)	32	8.713	**0.003**
	Male	34 (49.28)	3 (60.00)	37		
Total		54	15	69		
Obese	Female	8 (11.59)	8 (25.00)	16	8.597	**0.003**
	Male	17 (24.64)	1 (20.00)	18		
Total		25	9	34		
Column total		138	37	175		

Bold indicates statistically significant results (*p* < 0.05).

Table 3 presented non statically significant frequency differences between investigated individuals who reported and those who did not report thyroid diseases in the two 2D:4D digit ratio categories (below 1—male pattern, above or equal 1—female pattern) for the both hands. Odds ratio showed that female hand pattern in the right hand reduced the risk of thyroid diseases for the both sexes. In contrary to right hand for the left hand the results were contradictory, female hand pattern was a risk factor of thyroid diseases for both sexes, however the *p* value did not allow to interpret this result as a statistically significant.

**Table 3:** Frequency differences between investigated individuals who reported and those who did not report thyroid diseases in the 2D:4D digit ratio categories (below 1—male pattern, above or equal 1—female pattern) for the both hands.

Sex	Thyroid diseases N	2D4D (R) < 1 N (%)	2D4D (R) > = 1 N (%)	Row	Chi^2^	*p*	OR	CI (95%)	
Female	No	42 (24.00)	27 (15.43)	69 (39.43)	0.025	0.876	0.933	0.394	2.214
	Yes	20 (11.43)	12 (6.86)	32 (18.29)					
Total		62 (35.43)	39 (22.29)	101 (57.71)					
Male	No	48 (27.43)	21 (12.00)	69 (39.43)	0.243	0.622	0.571	0.061	5.424
	Yes	4 (2.29)	1 (0.57)	5 (2.86)					
Total		52 (29.71)	(12.57)	74 (42.29)					
Column total		114 (65.14)	61 (34.86)	175					

Sex	Thyroid diseases N (%)	2D4D (L) < 1	2D4D (L) > = 1	Row	Chi^2^	*p*	OR	CI (95%)	
Female	No	39 (22.29)	30 (17.14)	69 (39.43)					
	Yes	15 (8.57)	17 (9.71)	32 (18.29)	0.818	0.366	1.473	0.635	3.419
Total		54 (30.86)	47 (26.86)	101 (57.71)					
Male	No	43 (24.57)	26 (14.86)	69 (39.43)	0.011	0.918	1.103	0.173	7.042
	Yes	3 (1.71)	2 (1.14)	5 (2.86)					
Total		46 (26.29)	28 (16.00)	74 (42.29)					
Column Total		100 (54.14)	75 (42.86)	175					

The Spearman correlations presented in the Table 4 showed statistically significant results between BMI and WHR among all investigated individuals. BMI was negatively correlated with 2D:4D digit ratio (L) and BMI among males who had thyroid diseases. Among healthy women age was positively correlated with BMI and WHR. Only among males who had thyroid diseases there were negative correlation between the 2D:4D digit ratio (R) and age and between the 2D:4D digit ratio (L) and BMI.

**Table 4:** Association between age, BMI, WHR versus 2D:4D finger ratio for both hands separately for healthy and ill man and women.

Pair of variables	Females without thyroid diseases N = 69		Males without thyroid diseases N = 69		Females with thyroid diseases N = 32		Males with thyroid diseases N = 5	
	Spearman R	*p*-value	Spearman R	*p*-value	Spearman R	*p*-value	Spearman R	*p*-value
BMI box cox & WHR	0.811	**< 0.001**	0.819	**< 0.001**	0.749	**< 0.001**	0.900	**0.037**
BMI box cox & 2D4D P	0.047	0.701	− 0.099	0.420	0.133	0.469	− 0.400	0.505
BMI box cox & 2D4D L	0.018	0.883	− 0.037	0.761	0.108	0.558	− 0.900	**0.037**
BMI box cox & Age	0.470	**< 0.001**	0.120	0.325	0.266	0.141	0.300	0.624
WHR & 2D4D P	0.013	0.918	− 0.112	0.360	− 0.015	0.939	− 0.700	0.188
WHR & 2D4D L	− 0.031	0.803	− 0.065	0.593	0.048	0.801	− 0.800	0.104
WHR & Age	0.422	**< 0.001**	0.168	0.169	0.202	0.284	0.500	0.391
2D4D P & 2D4D L	0.653	**< 0.001**	0.404	**0.001**	0.435	**0.013**	0.300	0.624
2D4D P & Age	0.137	0.261	− 0.167	0.170	− 0.259	0.152	− 0.900	**0.037**
2D4D L & BMI box cox	0.018	0.883	− 0.037	0.761	0.108	0.558	− 0.900	**0.037**
2D4D L & Age	0.027	0.823	0.025	0.839	0.030	0.872	− 0.100	0.873
Age & 2D4D L	0.027	0.823	0.025	0.839	0.030	0.872	− 0.100	0.873

Bold indicates statistically significant results (*p* < 0.05).

**Figure 1:**
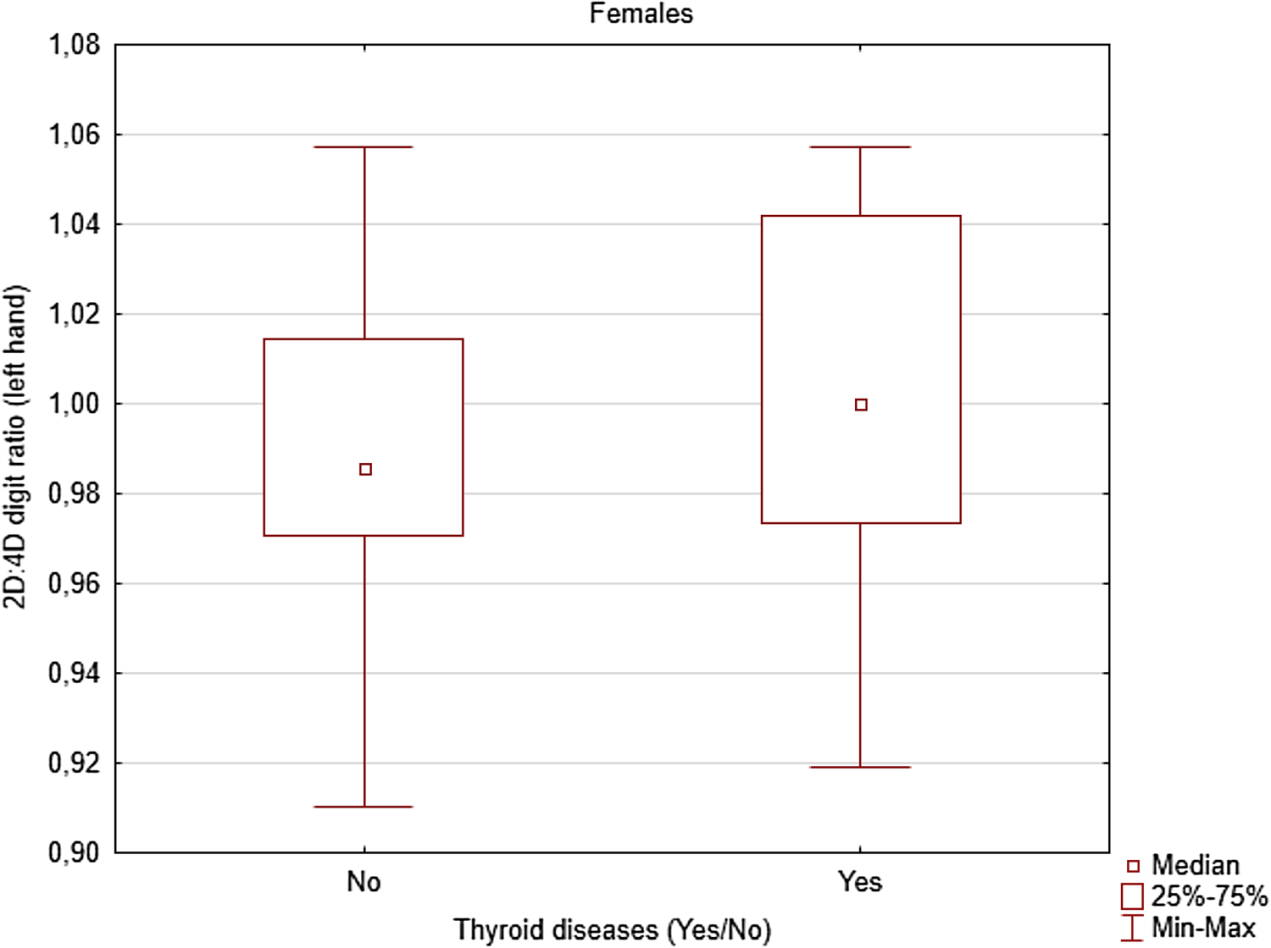
Differences in 2D:4D (L) among females who had thyroid diseases and healthy ones (t =  − 2.105; *p* = 0.038; d = 0.707).

BMI, age and WHR were not statistically significantly different among individuals who had thyroid diseases and healthy females and males (Table 5). Moreover, the 2D:4D digit ratio for both hands were not statistically significant among men who have thyroid disease and healthy one, but also for 2D:4D (R) among women have thyroid disease and healthy one. The differences between females who had thyroid diseases and healthy ones were observed only in the case of 2D:4D digit ratio (left hand). The women who had thyroid disease had higher 2D:4D digit ratio (left hand) (mean = 1.004; SD = 0.036) than healthy ones (mean = 0.989; SD = 0.030) (t = − 2105; *p* = 0.038; d = 0.707) (Table 5, Fig. 1).

**Table 5:** Differences in age, BMI, 2D:4D (R), 2D:4D (L) among individuals who had thyroid diseases and healthy one separately for females and males.

Sex	Thyroid diseases	Variables						t	*p*	d
		Means	N	SD	Q25	Median	Q75			
Females	**Age**									
	No	51.159	69	12.582	43.000	49.000	60.000	− 0.675	0.501	0.195
	Yes	52.969	32	13.094	42.500	52.000	65.000			
	**BMI (after Box–Cox transformation)**									
	No	1.034	69	0.008	1.030	1.033	1.038	− 2.594	**0.011**	0.470
	Yes	1.038	32	0.009	1.031	1.038	1.044			
	**WHR**									
	No	0.928	69	0.062	0.880	0.920	0.960	− 1.601	0.113	0.572
	Yes	0.953	32	0.108	0.860	0.950	1.030			
	**2D4D digit ratio (right hand)**									
	No	0.980	69	0.033	0.958	0.986	1.000	− 0.916	0.362	0.257
	Yes	0.986	32	0.038	0.961	0.985	1.021			
	**2D4D digit ratio (left hand)**									
	No	0.989	69	0.030	0.971	0.986	1.014	− 2.105	**0.038**	0.707
	Yes	1.004	32	0.036	0.973	1.000	1.042			
Males	**Age**									
	No	50.188	69	13.905	38.000	47.000	61.000	− 0.868	0.388	0.389
	Yes	55.800	5	14.923	45.000	47.000	70.000			
	**BMI (after Box–Cox transformation)**									
	No	1.039	69	0.008	1.033	1.038	1.043	0.147	0.884	0.133
	Yes	1.038	5	0.007	1.034	1.041	1.042			
	**WHR**									
	No	0.958	69	0.092	0.890	0.960	1.020	− 1.188	0.239	0.605
	Yes	1.008	5	0.072	0.950	1.010	1.040			
	**2D4D digit ratio (right hand)**									
	No	0.975	69	0.035	0.951	0.974	1.000	− 0.868	0.834	0.452
	Yes	0.956	5	0.048	0.925	0.948	0.975			
	**2D4D digit ratio (left hand)**									
	No	0.980	69	0.031	0.963	0.986	1.000	1.114	0.269	0.088
	Yes	0.977	5	0.037	0.959	0.960	1.000			

Bold indicates statistically significant results (*p* < 0.05).

The regression models explaining variability of 2D:4D (R) and (L) were performed separately for males and females. The following variables were included in the model: age, BMI, WHR and occurrence of the thyroid diseases. Neither for males nor for females' models for each hand were statistically significant (Supplementary data).

## Discussion

Among plenty of studies which discussed the association between second to third digit ratio and some of the human traits or diseases^27–30^, there is a lack of studies which investigated an association with thyroid diseases. Only Tabachnik et al.^19^ found that women with higher 2D:4D had more frequently thyroid disorders. Our results are partially in line with Tabachnik’s outcomes, as we also showed that females with higher 2D:4D (left hand) more commonly had thyroid diseases. Interestingly, we did not find it for the right hand. Some studies underline that right hand is more representative for digit ratio calculation^31^.

The relationship between prenatal programming of thyroid functions and sex hormones remains unclear, however Wilcoxon and Redei^20^ claimed that thyroid function is programmed in utero and might be regulated by sex steroids in the adulthood.

Thyroid hormones are transported by specific protein: thyroxine-binding globulin (TBG). Estrogen causes increase in the concentration of TBG which leads to increase concentration thyroid hormone (T4). However, there are evidences that thyroid disorders may be a consequence of direct estrogen's influence on hypothalamic-pituitary-thyroid axis (HPT) and unrelated to TBG e.g. elevated TSH^32,33^.

There are studies which consider thyroid diseases and sex hormone proportions. Elevated estrogen levels seem to occur more commonly among individuals with hypothyroidism^10^, that is less frequently observed in men than in women^11^.

In contrary, some studies presented the link between the occurrence of the thyroid diseases and PCOS, that is connected with estrogen, progesterone and androgens imbalance. Some of the studies showed that elevated level of androgens may relate to PCOS and hypothyroidisms^10,11,34,35^. Similarly, female with lower estrogen level during menopausal period more frequently had hypothyroidism as Abdel-Dayem, M.M. and M.S. Elgendy showed in rats' studies^34^.

Additionally, there are some suspicions that elevated testosterone level might be associated with excess of thyroid functions. The levels of testosterone in Chinese male patients with TPP—thyreotoxic periodic paralysis (elevated thyroid hormone production) was higher compared to those with only hyperthyroidism^36^.

Among adults there was not observed correlation between age and 2D:4D finger ratio^37^, although we presented the negative correlation between age and 2D:4D finger ratio but only for the right hand among man who had thyroid diseases, thus more study is needed in this area.

We did not replicate the results of the study performed by Bagepally et al.^22^ who presented that higher 2D:4D finger ratio is associated with elevated BMI. We showed only dimorphic differences—women had statistically significantly lower BMI and less frequently had overweight and obesity than men, that is in line with other studies around the world^38,39^. Women are more familiar with healthy lifestyle than men that results in lower BMI among women than in men^40^. However, worldwide the prevalence of obesity is higher among women than in men independently on country development status^41–43^. These results lead to the conclusion that extreme cases of excessive body mass are more common among women but simultaneously the women are more likely to take care of their health status. Probably the obesity among women is more frequently associated with secondary obesity stemming from comorbidities.

Additionally, we did not find any differences in BMI among individuals who had the thyroid diseases and healthy ones, that need more precise studies in the future.

### Limitations

Our study importantly underlined the association between thyroid disorders and prenatal exposition to sex hormones. Nevertheless, the main limitation of the study was small number of subjects, which did not allow the use of multivariate statistical methods. Additionally, we did not include information about duration of hypothyroidism and Hashimoto diseases. Moreover, we did not include information about menopause. Finally, besides some limitations that was the first study which tackled the problem of association between thyroid disorders and 2D:4D digit ratio which research sheds new light on. The current research seems to be a novel and promising for further detailed investigation.

## Conclusions

The women with thyroid diseases tend to have higher 2D:4D digit ratio for the left hand. The association between thyroid diseases and 2D:4D digit ratio is tentative, however prenatal exposition to estrogens seems to increase the probability of thyroid diseases in the future life.

## Supplementary Information

Supplementary Table S1.
