# Supplementary material for: Thyroid diseases and second to fourth digit ratio in Polish adults
Source: Sci Rep. 2021 Sep 23;11:18979. doi: 10.1038/s41598-021-98436-4 (PMC8460635; doi:10.1038/s41598-021-98436-4)
Supplement: Supplementary file 1 — Supplementary Table S1. [file 41598_2021_98436_MOESM1_ESM.docx]

Supplementary data

Supplementary Table. The regression models explaining variability of 2D:4D (R) and (L), separately for males and females.

| Man N=74 | | | | | | | | | | Women N=101 | | | | | | | | |
| --- | --- | --- | --- | --- | --- | --- | --- | --- | --- | --- | --- | --- | --- | --- | --- | --- | --- | --- |
|  | 2D:4D digit ratio (L) | | | | | | | | | | | | | | | | | |
|  | b* , | Std.Err of b* , | b | Std.Err of b | t(69) | p-value | R2 | F | p | b* | Std.Err.  of b* | b | Std.Err.  of b | t(96) | p-value | R2 | F | p |
| Intercept |  |  | 1.493 | 0.829 | 1.802 | 0.076 | <0.001 | 0.259 | 0.903 |  |  | 1.136 | 0.597 | 1.902 | 0.060 | 0.006 | 1.142 | 0.342 |
| Age | 0.076 | 0.122 | 0.000 | 0.000 | 0.621 | 0.536 |  |  |  | 0.079 | 0.110 | 0.000 | 0.000 | 0.719 | 0.474 |  |  |  |
| BMI box cox | -0.125 | 0.206 | -0.514 | 0.851 | -0.604 | 0.548 |  |  |  | -0.043 | 0.165 | -0.162 | 0.622 | -0.260 | 0.795 |  |  |  |
| WHR | 0.036 | 0.210 | 0.013 | 0.073 | 0.172 | 0.864 |  |  |  | 0.025 | 0.157 | 0.010 | 0.063 | 0.158 | 0.875 |  |  |  |
| Occurence of the thyroid diseases (Yes(1)vs. No(0)) | -0.040 | 0.124 | -0.005 | 0.016 | -0.320 | 0.750 |  |  |  | 0.200 | 0.105 | 0.014 | 0.007 | 1.909 | 0.059 |  |  |  |
|  | 2D:4D digit ratio (R) | | | | | | | | | | | | | | | | | |
|  | b* | Std.Err.  of b* | b | Std.Err.  of b | t(69) | p-value | R2 | F | p | b* | Std.Err.  of b* | b | Std.Err.  of b | t(96) | p-value | R2 | F | p |
| Intercept |  |  | 2.066 | 0.883 | 2.340 | 0.022 | 0.060 | 2.159 | 0.022 |  |  | 0.565 | 0.657 | 0.860 | 0.392 | <0.00.1 | 0.368 | 0.831 |
| Age | -0.213 | 0.116 | -0.001 | 0.000 | -1.834 | 0.071 |  |  |  | -0.069 | 0.112 | 0.000 | 0.000 | -0.617 | 0.539 |  |  |  |
| BMI box cox | -0.224 | 0.196 | -1.034 | 0.906 | -1.141 | 0.258 |  |  |  | 0.105 | 0.167 | 0.431 | 0.685 | 0.629 | 0.531 |  |  |  |
| WHR | 0.026 | 0.200 | 0.010 | 0.078 | 0.131 | 0.896 |  |  |  | -0.053 | 0.159 | -0.023 | 0.070 | -0.332 | 0.740 |  |  |  |
| Occurence of the thyroid diseases(Yes(1)vs. No(0)) | -0.116 | 0.118 | -0.016 | 0.017 | -0.986 | 0.328 |  |  |  | 0.089 | 0.106 | 0.007 | 0.008 | 0.839 | 0.404 |  |  |  |
